# Supplementary material for: Dendronized fluorosurfactant for highly stable water-in-fluorinated oil emulsions with minimal inter-droplet transfer of small molecules
Source: Nat Commun. 2019 Oct 4;10:4546. doi: 10.1038/s41467-019-12462-5 (PMC6778136; doi:10.1038/s41467-019-12462-5)
Supplement: Supplementary file 1 — Supplementary Information [file 41467_2019_12462_MOESM1_ESM.pdf]

Supplementary Information (SI)

Dendronized fluorosurfactant for highly stable water-in-fluorinated oil emulsions with minimal inter-droplet transfer of small molecules

Chowdhury et al.

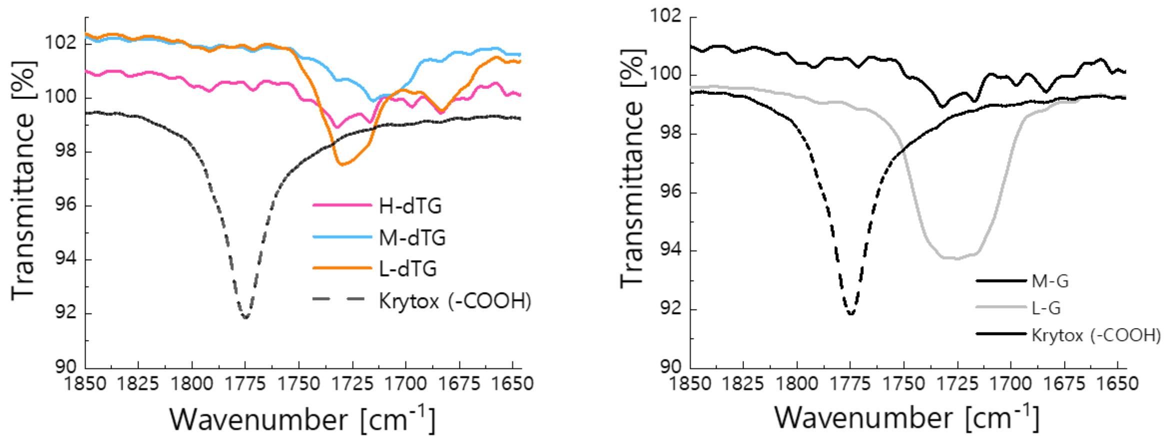

Supplementary Figure 1. Characterization of the synthesized surfactants. We record FTIR spectra from viscous products of dendritic tri-glycerol (dTG)-based surfactants (left) and mono-glycerol (G)-based surfactants (right). When dTG- or G-precursor is used to functionalize Krytox of either high (H), medium (M), or low (L) molecular weight (MW), then either H-dTG, M-dTG, L-dTG, M-G or L-G is created. The unmodified carboxylic acid band of low molecular weight Krytox precursor shows strong peak at 1775 cm^{-1} on both figures (left and right) depicted by dotted lines (gray). The amide (-NH-CO-) stretching peaks, H-dTG (solid red line), M-dTG (solid green line), L-dTG (solid yellow line), M-G (solid black line), and L-G (solid gray line), from 1710-1740 cm^{-1} confirm the functionalization of low, medium, and high molecular weight Krytox terminuses with either dTG- or G-polar group.

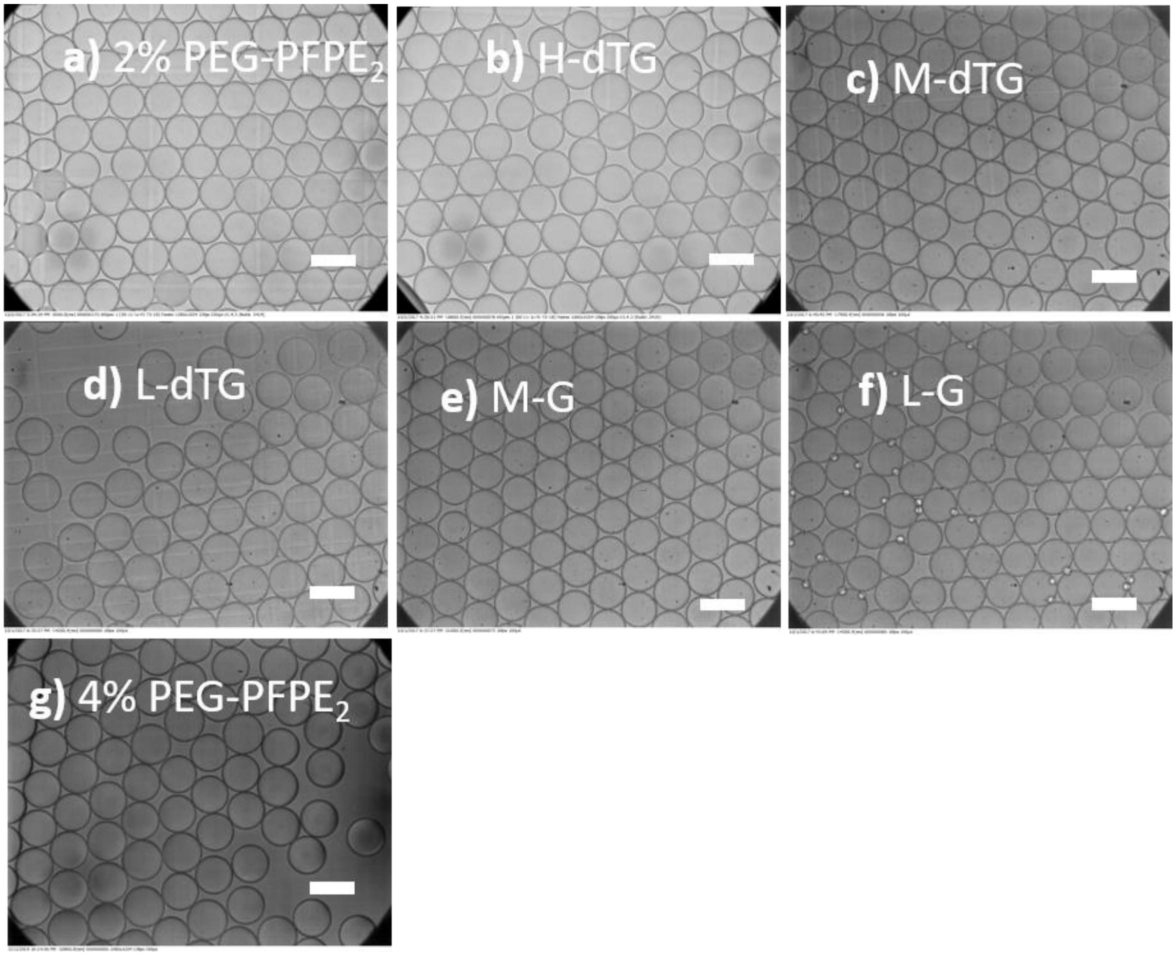

Supplementary Figure 2. Thermostability of the droplets before PCR thermocycle (pre-PCR). We use polydimethylsiloxane (PDMS) based microfluidic device to generate PCR-reagents carrying monodisperse droplets that are stabilized with 2% PEG-$PFPE_2$ surfactant (**a**), H-dTG surfactant (**b**), M-dTG surfactant (**c**), L-dTG surfactant (**d**), M-G surfactant (**e**), L-G surfactant (**f**), and 4% PEG-$PFPE_2$ surfactant (**g**). PEG-$PFPE_2$ is a reference surfactant in this study that uses PEG600 as a polar head group, which is a commercially available tri-block copolymer surfactant, EA surfactant, unlike our dendronized di-block surfactants. Droplets produced using the dTG, the G, and the PEG600-based surfactants showed no merging during >24h incubation at 4^oC. Scale bar, 100 μm.

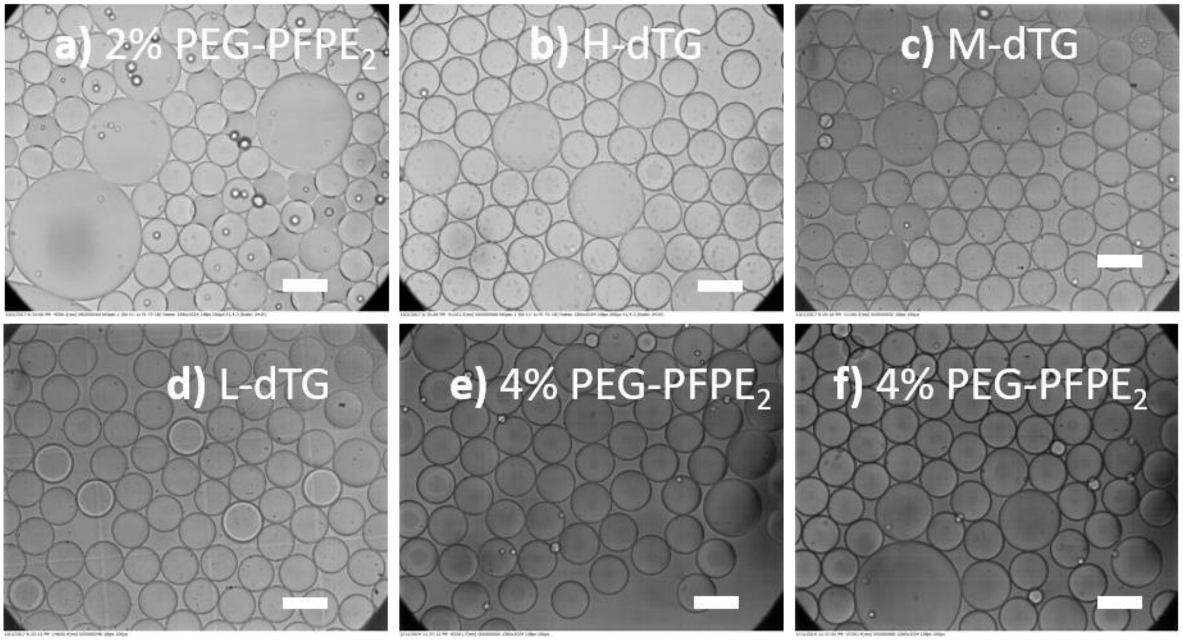

Supplementary Figure 3. Thermostability of the droplets after PCR thermocycle (post-PCR). We run a total of 35 cycles of PCR, which involves repeated thermal cycling from ~60°C to 98°C, enabling nucleic acid amplification. During PCR, droplets stabilized with commercially available PEG600-based surfactant (2% PEG-PFPE$_2$) showed substantial merging (**a**). H-dTG and M-dTG surfactants stabilized droplets showed some merging (**b** and **c** respectively). Surprisingly, the L-dTG surfactant stabilized droplets showed almost no merging (**d**). However, droplets stabilized with a higher concentration (4%) of PEG-PFPE$_2$ surfactant, performed as well as the H-dTG surfactant stabilized droplets (**e**-**f**). In contrast, droplets generated using mono-glycerol (G)-based surfactants (M-G, L-G) merged completely during the PCR, suggesting the lack of more polar hydroxy groups. Scale bar, 100 μm.

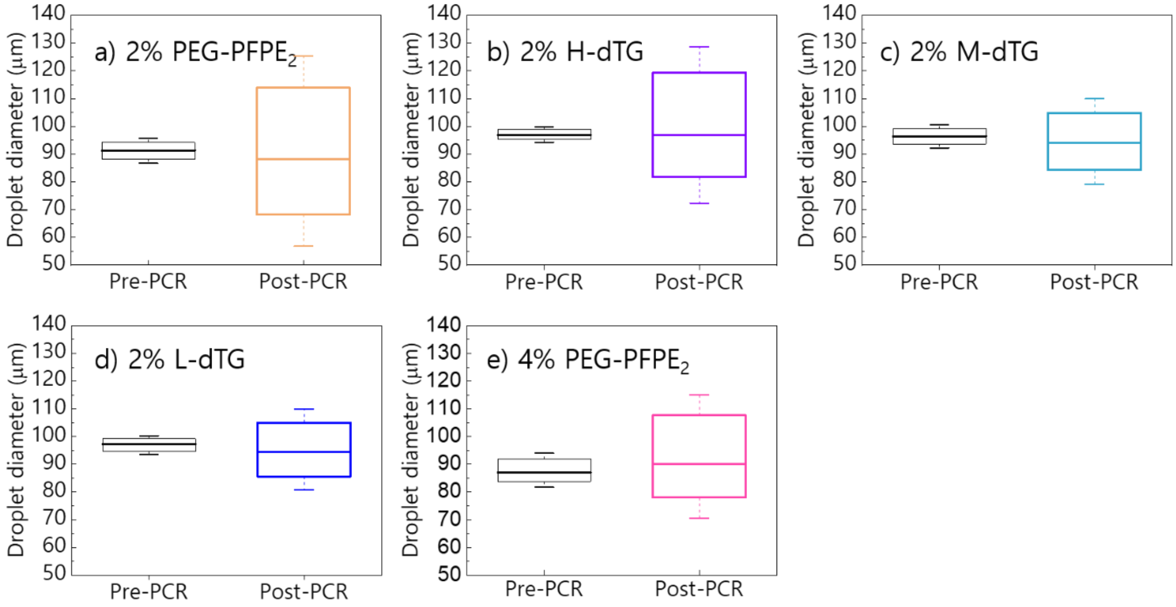

Supplementary Figure 4. Box-plot of droplet size distribution pre-and post-PCR. We run a total of 35 cycles of PCR, image a fraction of the droplets, and use a purpose-written Matlab script to analyze droplet size distribution. n = ~100 droplets for Pre-PCR size distribution analysis and n= ~200-400 droplets for Post-PCR size distribution analysis**.** The box plots represent the median (center line), mean ± s.d. (box), and mean ± 1.5 x s.d. (whisker). Source data of Supplementary Figures 4a-4e are provided as a Source Data file.

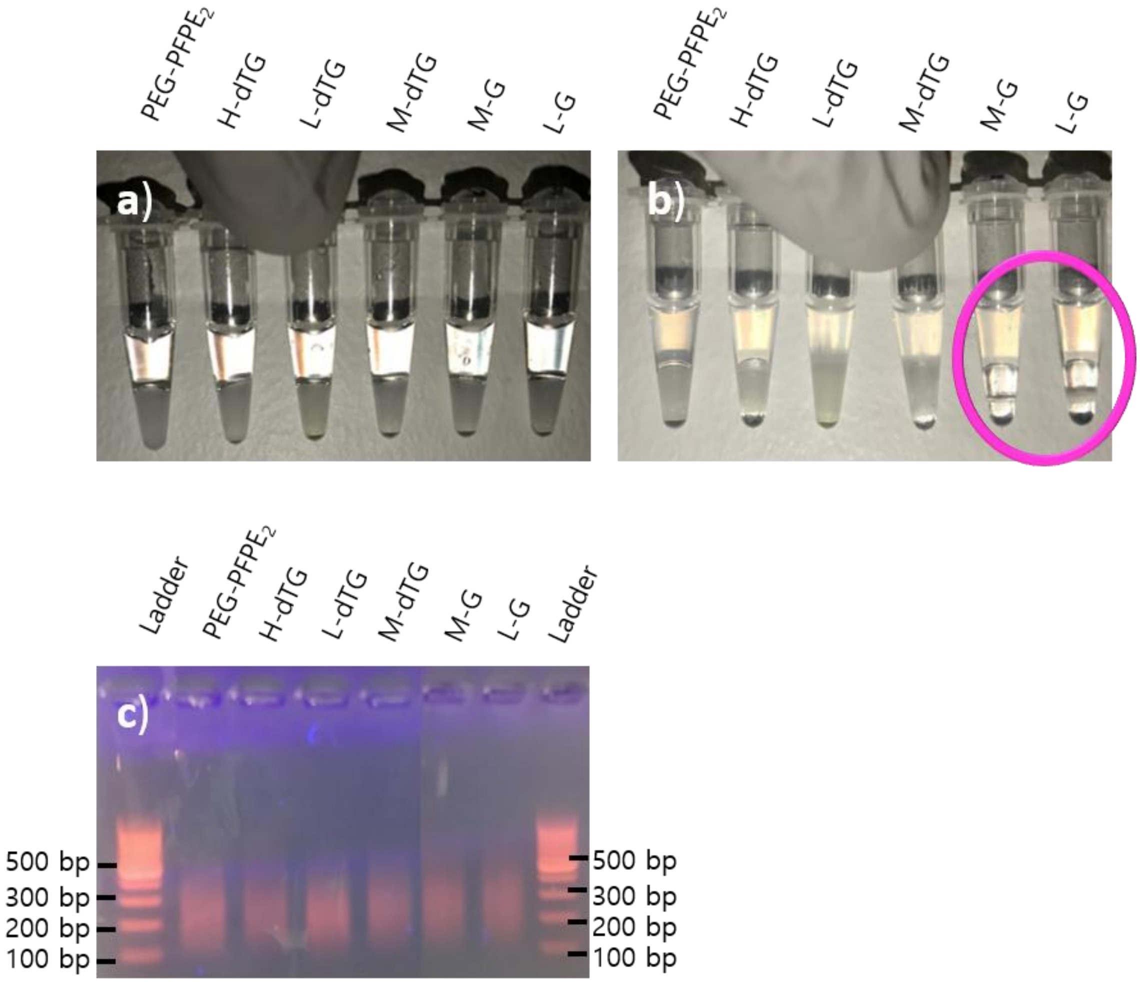

Supplementary Figure 5. Emulsion droplet integrity pre- and post-PCR, and analysis of PCR amplification product. a, For PCR thermocycling, we generate PCR-mix-containing droplets stabilized by six different surfactants (from left to right: 2% PEG-PFPE$_2$, H-dTG, L-dTG, M-dTG, M-G, L-G). We collect the emulsion droplets (gray bottom phase) in Eppendorf tubes and then add heavy mineral oil (bright phase) to prevent evaporation during PCR cycling. Prior to cycling, droplet emulsions are stable, as indicated by homogeneous droplet layer underneath the layer of mineral oil. **b,** Analysis of the post-PCR emulsions reveals that droplets made with M-G and L-G surfactants are not stable, as indicated by the water layer, resulting from merged droplets, below the mineral oil (see tubes regions circled in red). PEG-PFPE$_2$, H-dTG, and M-dTG surfactants performed better in PCR, as shown by the small water layer

between the mineral oil and emulsion droplet layers. Notably, no droplet merging is seen in the L-dTG stabilized emulsion droplets, indicating suitability of L-dTG surfactants in thermal-cycling applications. **c,** We electrophorese 10 µl of the 40 µl aqueous phase collected from breaking the droplets on 2% agarose gel to confirm that surfactants are free from PCR-inhibitors. 0.5 µg of 100 bp ladder was loaded into the first and last lanes. None of the surfactants inhibited PCR, as indicated by the strong amplification product in each sample lane. The products are a mixture of molecular weights because the phage genomic DNA is sheared randomly by Nextera Tagmentation and amplified using primers to the added tag DNA sequence. Each 40 µl PCR contained 16 pg of input DNA; thus, the unamplified product is not visible on this type of gel (data not shown). Source data of Supplementary Figure 5c is provided as a Source Data file.

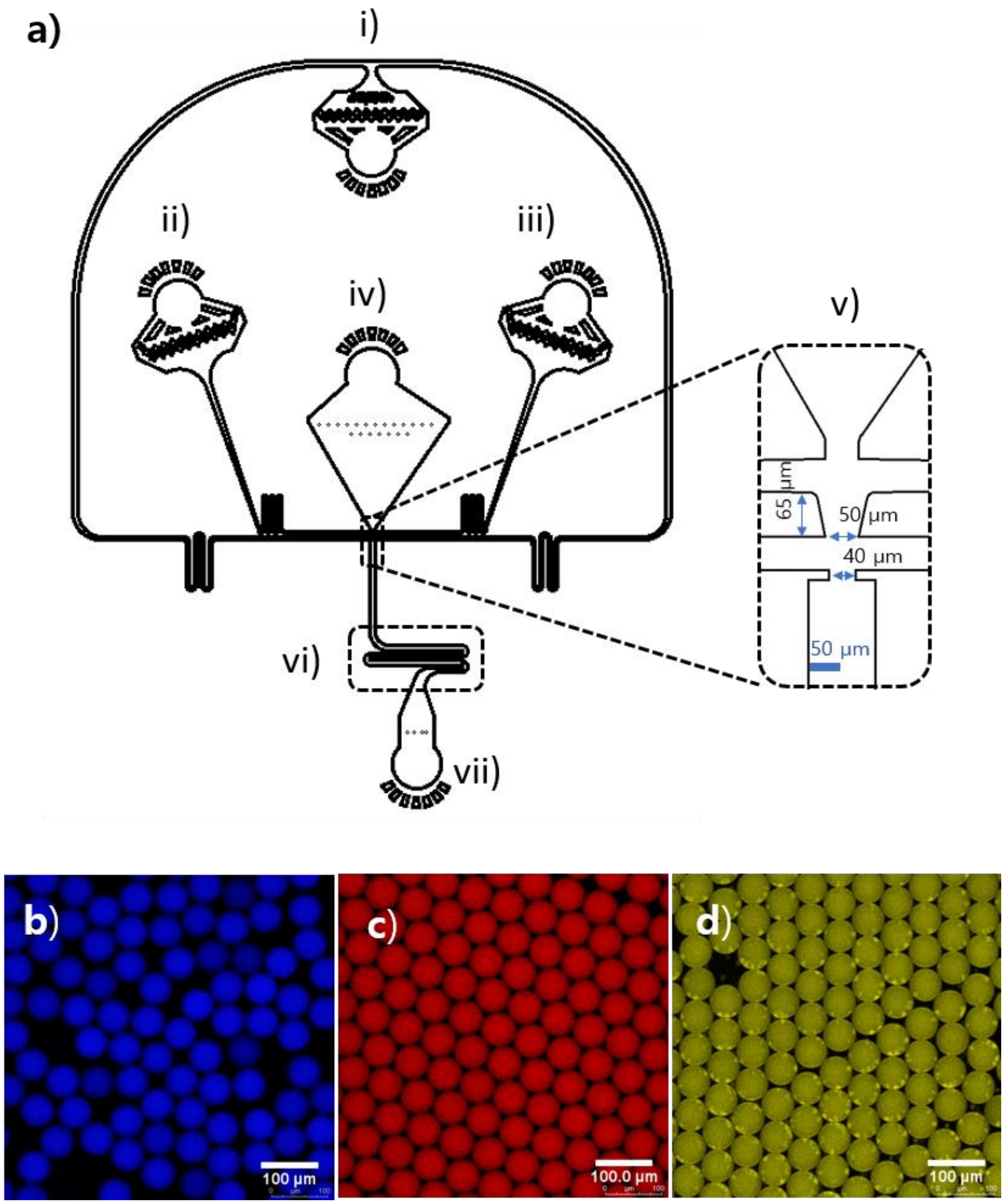

Supplementary Figure 6. Single drop making microfluidic device for click-chemistry based microgel template preparation in droplets stabilized by dendritic tri-glycerol (dTG)-based surfactants. a, This device produces monodisperse droplets. Inlet for surfactant-containing HFE7500 oil continuous phase (i). Inlets for different polymer precursor solutions (ii and iii). Additional inlet to encapsulate cells or to inject aqueous medium carrying different solutes (iv). Flow focusing junctions to make micrometer sized microgel droplets. The cross-section of the flow-focusing nozzle at

the pinch-off area is 40 µm x 50 µm (v). Delay line keeps droplets spaced while surfactant molecules orient at oil-water interface (vi). Outlet for the polymer precursors containing droplets (vii). This PDMS device allows us to fabricate microgels with 20% (w/v) polyanionic dendritic polyglycerol sulfate azide (dPGS-N_3), 20% (w/v) homobifunctional PEG dicyclooctyne (PEG-DIC), and 1% (w/v) of mono-fluorophore functionalized PEG-DIC that undergo SPAAC reaction in droplets, creating cross-linked networks[1, 2]. Flow rates of 750 µl/h for the surfactant-containing HFE7500 continuous phase oil, 67 µl/h for the dPGS-N_3 aqueous phase, 133 µl/h for the PEG-DIC aqueous phase, and 40 µl/h for an additional aqueous phase containing only PBS medium create droplets of ~50 µm diameter. The overall polymer concentration in the aqueous phase is ~17% (w/v). We click-label the microgels with blue (3-azido-7-hydroxycoumarin, Jena Bioscience), red (Sulfo-Cy5-Azide, Jena Bioscience), and yellow (5/6-Sulforhodamine B-PEG_3-Azide, Jena Bioscience) fluorophores when the droplets are stabilized with H-dTG (**b**), M-dTG (**c**), and L-dTG (**d**) surfactants respectively. Long-term or short-term incubation of the droplets shows no inter-microgels cross-linking, suggesting that dendritic tri-glycerol (dTG)-based H-dTG, M-dTG, and L-dTG surfactants can stabilize viscous aqueous droplets and allow formation of cross-linked polymer networks.

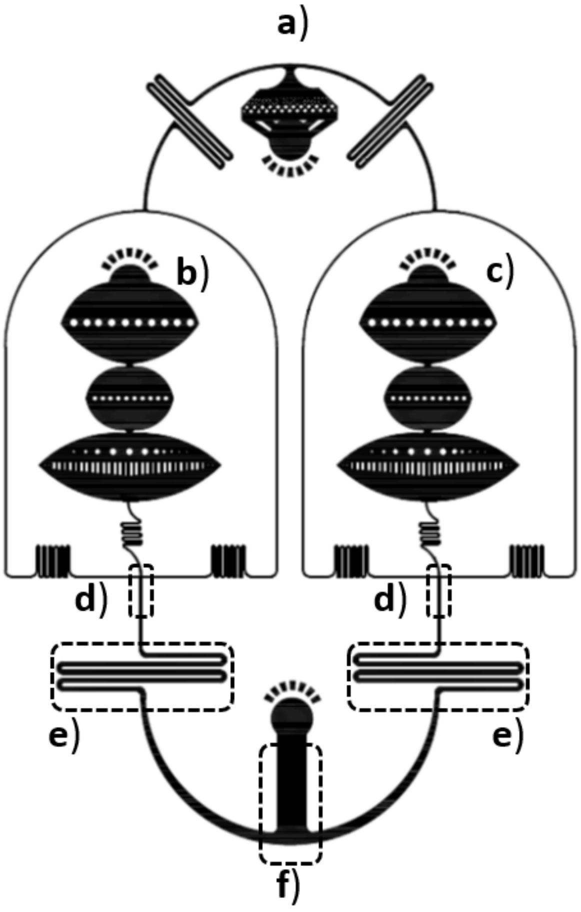

Supplementary Figure 7. Design of parallel drop making microfluidic device. This device produces a mixture comprising equal amounts of two different populations of droplets. **a,** Inlet for surfactant-containing HFE7500 oil continuous phase. **b,** and **c,** Inlets for different aqueous phases. **d,** Flow focusing junctions to make micrometer sized aqueous droplets. **e,** Delay line keeps droplets spaced while surfactant molecules orient at oil-water interface. **f,** The two different droplet populations join and mix in this broad channel prior to exiting the device.

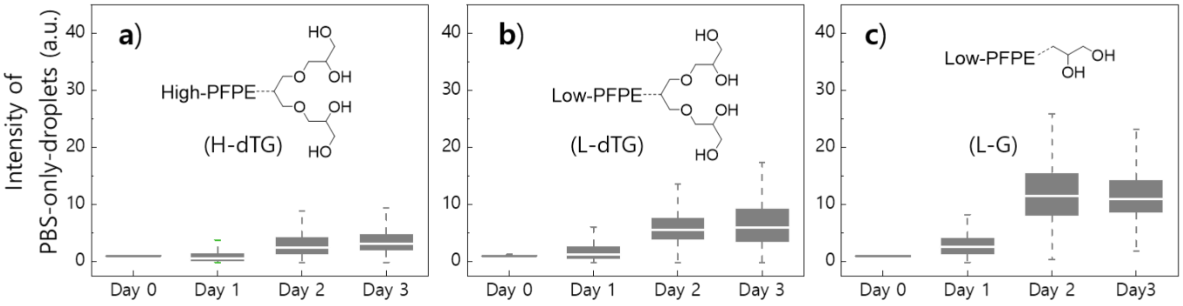

Supplementary Figure 8. Influence of long and short fluorinated tails and dense hydrogen bond network on inter-droplet diffusion. For each surfactant, we use a parallel drop maker (Supplementary Figure 7) to create a mixture comprising equal amounts of PBS-only-droplets and PBS+sodium fluorescein salt-containing droplets. We incubate these mixtures, take confocal images at the indicated time points, and then perform quantitative analysis of the fluorescence intensity of the 10 randomly selected PBS-only droplets. Box-plot shows that in droplets stabilized by H-dTG, there is almost no increase in green signal intensity after 24h incubation (**a**), while some increase was detected in L-dTG stabilized droplets (b), indicating that the loner fluorinated tail of H-dTG contributes to improved resistance to inter-droplet diffusions. To assess the effect of head groups capable of forming a dense hydrogen bond network, we compare L-dTG and L-G. Quantitatively, after 24h incubation, the fluorescence intensity of PBS-only drops stabilized by L-G, was, 1.5 times the intensity measured in PBS-only droplets stabilized by L-dTG surfactants, indicating that a dense hydrogen bond network at the oil-water interface also reduces leakage of small water-soluble molecules (compare panels **b** and **c**). In droplets stabilized by H-dTG, having long fluorinated tails and head groups capable of forming a dense hydrogen bond network, the fluorescent signal in PBS-only droplets was one third that seen for droplets stabilized by L-G, which has short fluorinated tails and head groups capable of only limited hydrogen-bonding. This indicates that long fluorinated tail groups and head groups capable of forming a dense hydrogen bond network can function together within the same surfactant molecule. The box plots represent the median

(center line), the interquartile range (box) and the non-outlier range (whiskers). Source data of Supplementary Figures 8a-8c are provided as a Source Data file.

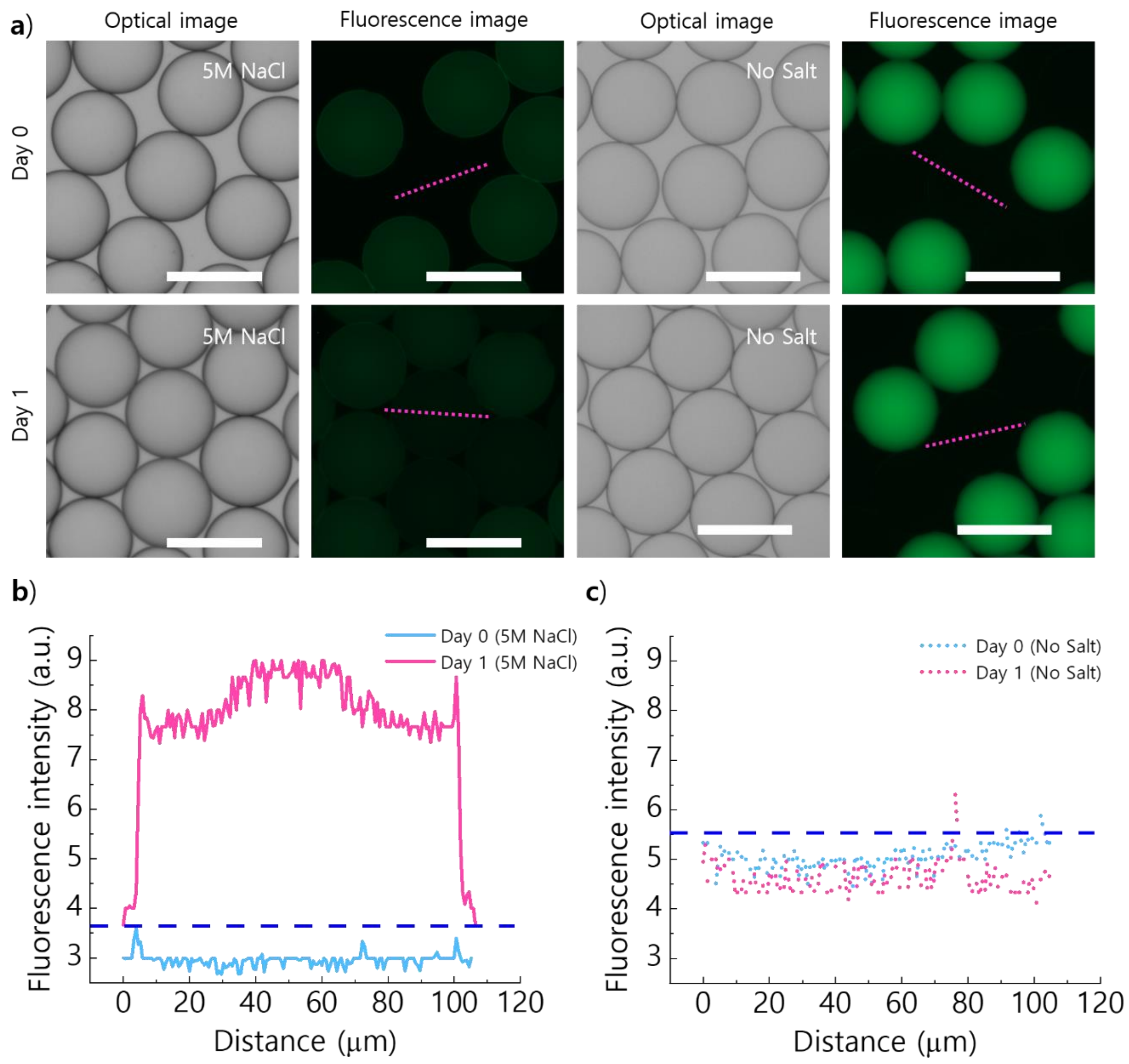

Supplementary Figure 9. Characterization of inter- and intra-molecular hydrogen bonds from dendritic tri-glycerol head groups. To test the contribution of hydrogen-donor activity of the hydroxyl groups in oligo-glycerol-based surfactants, we test if a high salt concentration, known to form ion-dipole interactions and disrupt inter-and intramolecular hydrogen bonding, decreases dye retention in droplets stabilized with the oligo-glycerol-based surfactant M-dTG. We use a parallel drop

maker (Supplementary Figure 7) to create a mixture comprising equal amounts of PBS-only-droplets and PBS+sodium fluorescein salt-containing droplets (No salt droplets), and a mixture comprising equal amounts of PBS+5M NaCl droplets and PBS+5M NaCl+sodium fluorescein salt-containing droplets (5M NaCl droplets). We incubate these mixtures, take fluorescence images at the indicated time points, and then use Image J Plot Profiling to perform quantitative analysis of the fluorescence intensity of the PBS-only droplets and PBS+5M NaCl droplets **(a-c)**. Scale bar, 100 μm. Note, the fluorescence emission intensity of sodium fluorescein salt is known to be reduced in the presence of high salt. Thus, our No Salt droplets have much higher overall intensity than the 5M NaCl droplets. However, this does not affect the interpretation of the data, as we separately compare the increase in fluorescence of the empty droplets from day zero to day one.

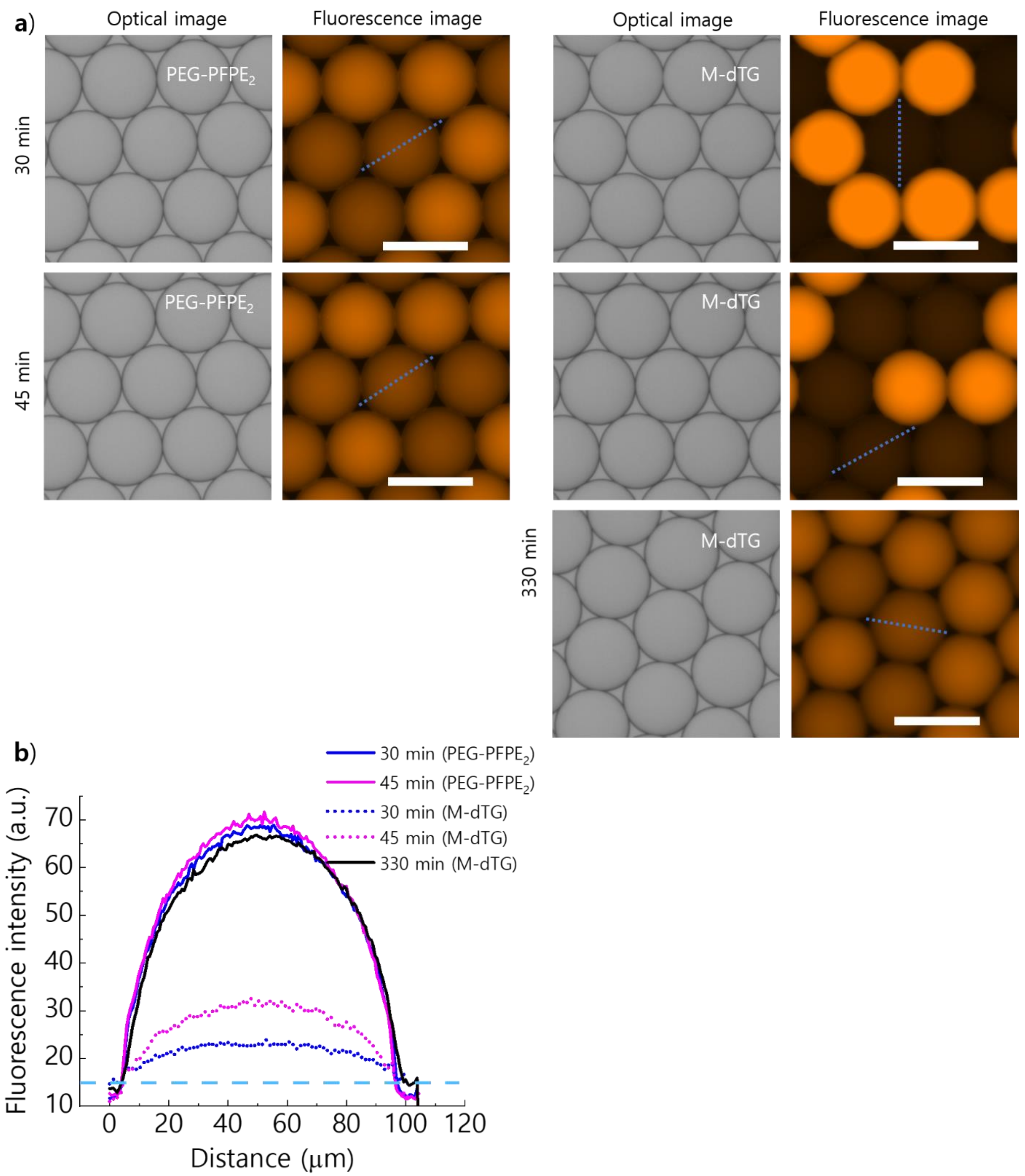

Supplementary Figure 10. Inter-droplet diffusion of resorufin dye is minimized by surfactant having head group capable of forming a dense hydrogen bond network. We use M-dTG surfactant, as we have shown that droplets stabilized with this surfactant retain sodium fluorescein dye better than droplets stabilized with other

oligo-glycerol based surfactants (see Fig. 3a in the main text). For comparison, we use droplets stabilized with commercially available PEG-$PFPE_2$ at 4% w/w, as this gives roughly equimolar concentration to 2% M-dTG. For each surfactant, we use a parallel drop maker (Supplementary Figure 7) to create a mixture comprising equal amounts of PBS-only-droplets and PBS+resorufin-containing droplets. We incubate these mixtures at room temperature, take fluorescence images at the indicated time points, and then use the Image J Plot Profiling tool to perform quantitative analysis of the fluorescence intensity of the PBS-only droplets. **a,** Micrographs of the resorufin-containing droplets demonstrates that resorufin transfer between M-dTG-stabilized droplets is much less than resorufin transfer between PEG-$PFPE_2$ stabilized droplets. Scale bar, 100 μm. **b,** Intensity profile of PBS-only droplets stabilized with PEG-$PFPE_2$ and M-dTG surfactants at the indicated time points. The corresponding fluorescence images can be seen in Supplementary Figure 10(a).

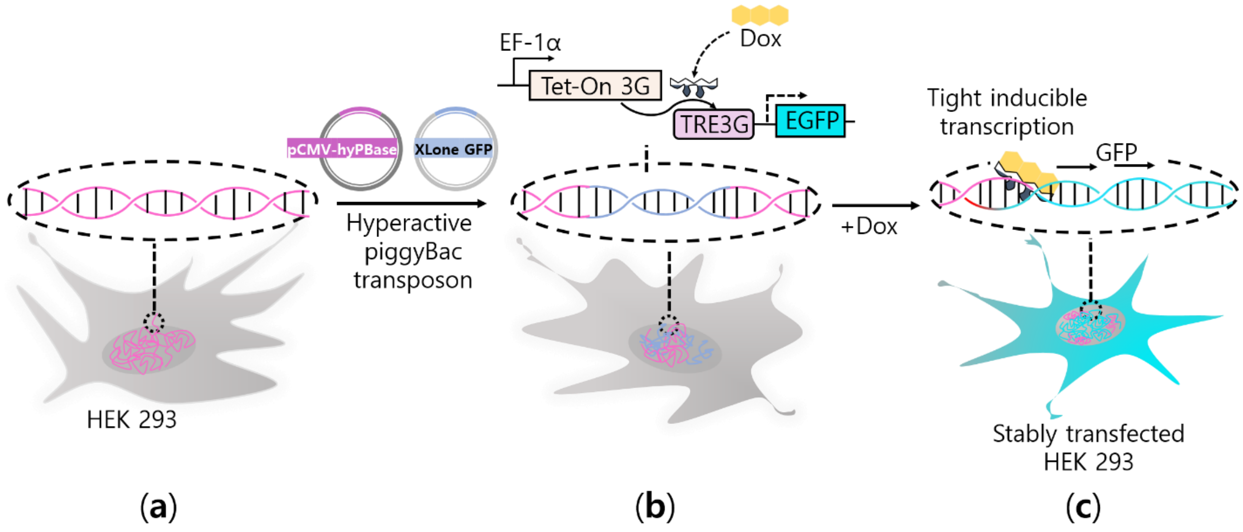

Supplementary Figure 11. Schematic representation of DOX-inducible stable cell line generation. a, HEK 293 cell line with native genome (red). **b,** Transposase from pCMV-hyPBase stably integrates XLone-GFP plasmid construct into the cell genome (light blue), generating DOX-sensitive reporter cell line (DOX-GFP-HEK 293) **c,** When DOX (yellow) binds to a trans-activator protein, cell produces green fluorescence protein (GFP).

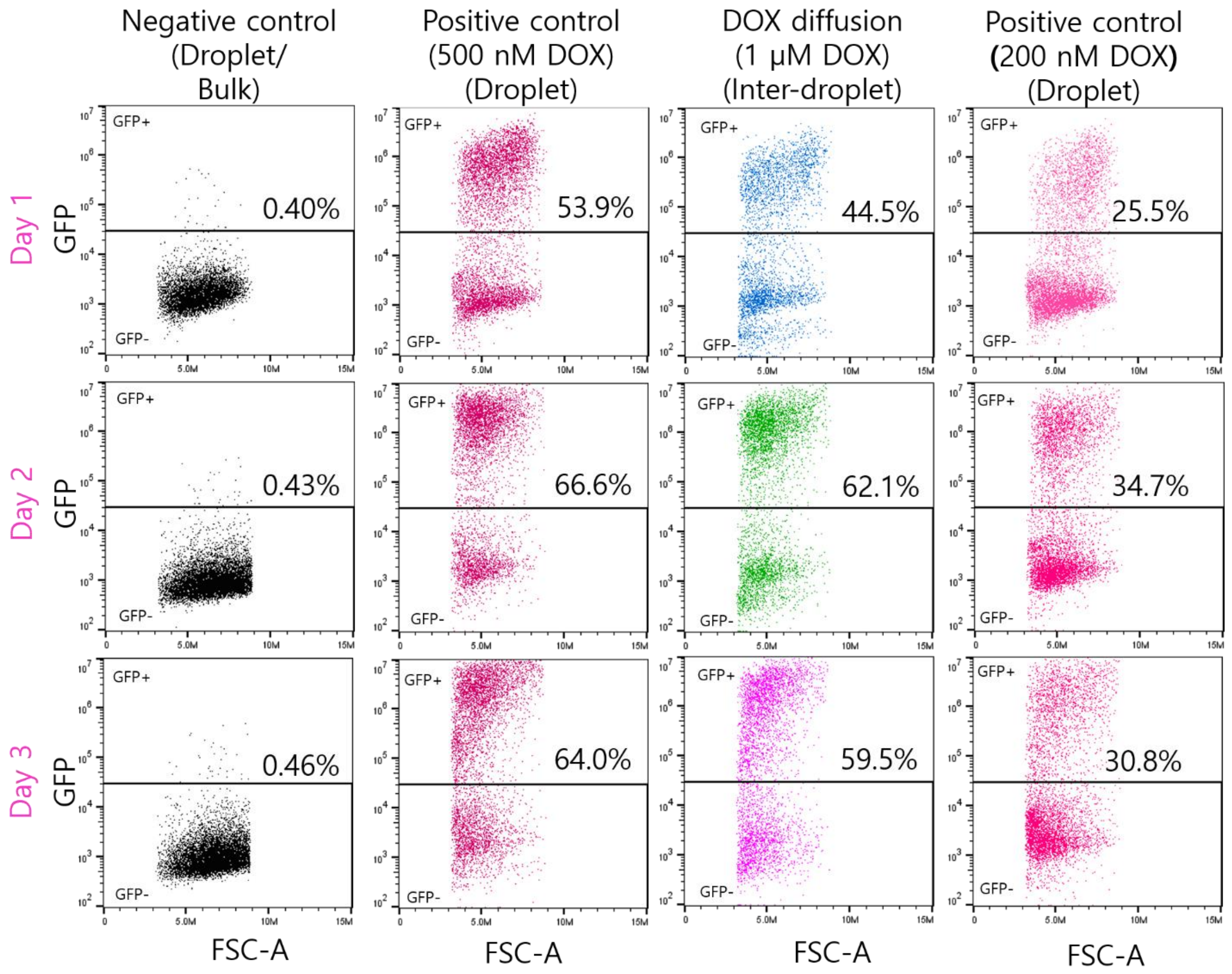

Supplementary Figure 12. DOX-inducible GFP-reporter cells to quantify drug transfer between PEG-PFPE$_2$ surfactant-stabilized droplets. We used 4% (w/w) PEG-PFPE$_2$ as a reference surfactant dissolved in HFE7500 oil and the parallel drop maker to generate homogenous mixtures in which 50% of the droplets contained DOX (at the indicated concentrations) and 50% contain no DOX. Due to Poisson distribution during cell encapsulation, ~40% of droplets that lack DOX contained at least one cell and the remaining ~10% of the droplets were empty. We incubated droplets at 37°C for the indicated times, isolated the cells from the droplets, and quantified the GFP+ cells using flow cytometry. GFP intensity is plotted against forward scatter-area (FSC-A). For the Positive Control (Droplet), all droplets, including those with cells, contained DOX at the indicated concentration.

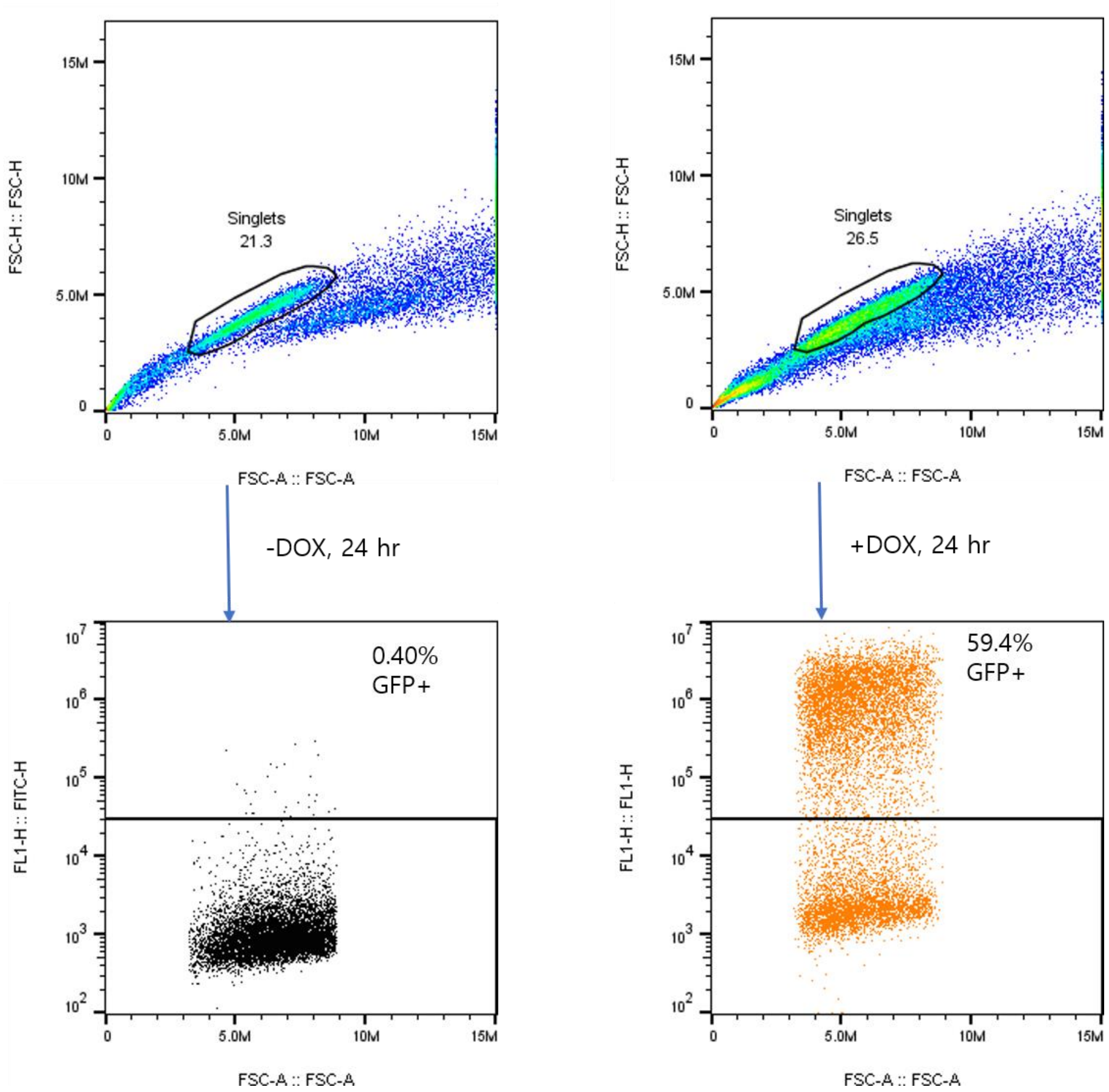

Supplementary Figure 13. Singlet cell population gating and comparison of GFP expression with and without DOX. Singlet cell population was selected from FSC-H/FSC-A gate to plot GFP intensity against forward scatter-area (FSC-A).

References

1. Dey P, Schneider T, Chiappisi L, Gradzielski M, Schulze-Tanzil G, Haag R. Mimicking of Chondrocyte Microenvironment Using In Situ Forming Dendritic Polyglycerol Sulfate-Based Synthetic Polyanionic Hydrogels. *Macromol. Biosci.* **16**, 580-590 (2016).

2. Steinhilber D, Rossow T, Wedepohl S, Paulus F, Seiffert S, Haag R. A microgel construction kit for bioorthogonal encapsulation and pH-controlled release of living cells. *Angew. Chem., Int. Ed. Engl.* **52**, 13538-13543 (2013).
